# Efficacy of a multimodal intervention strategy in improving hand hygiene compliance in a tertiary level intensive care unit

**DOI:** 10.4103/0972-5229.78215

**Published:** 2011

**Authors:** Ashu S. Mathai, Smitha E. George, John Abraham

**Affiliations:** **From:** Department of Anaesthesiolgy and Critical Care, Christian Medical College, Ludhiana, Punjab, India

**Keywords:** Hand hygiene compliance, intensive care unit, multimodal intervention

## Abstract

**Context:**

The role of hand hygiene in preventing health care associated infections (HCAIs) has been clearly established. However, compliance rates remain poor among health care personnel.

**Aims:**

a) To investigate the health care workers’ hand hygiene compliance rates in the intensive care unit (ICU), b) to assess reasons for non-compliance and c) to study the efficacy of a multimodal intervention strategy at improving compliance.

**Settings:**

A mixed medical–surgical ICU of a tertiary level hospital.

**Design:**

A before–after prospective, observational, intervention study.

**Materials and Methods:**

All health care personnel who came in contact with patients in the ICU were observed for their hand hygiene compliance before and after a multimodal intervention strategy (education, posters, verbal reminders and easy availability of products). A self-report questionnaire was also circulated to assess perceptions regarding compliance. Statistical analysis was done using *χ*^2^ test or Fisher exact test (Epi info software).

**Results:**

Hand hygiene compliance among medical personnel working in the ICU was 26% and the most common reason cited for non-compliance was lack of time (37%). The overall compliance improved significantly following the intervention to 57.36% (*P*<0.000). All health care worker groups showed significant improvements: staff nurses (21.48–61.59%, *P*<0.0000), nursing students (9.86–33.33%, *P*<0.0000), resident trainees (21.62–60.71%, *P*<0.0000), visiting consultants (22–57.14%, *P*=0.0001), physiotherapists (70–75.95%, *P*=0.413) and paramedical staff (10.71–55.45%, *P*< 0.0000).

**Conclusions:**

Hand hygiene compliance among health care workers in the ICU is poor; however, intervention strategies, such as the one used, can be useful in improving the compliance rates significantly.

## Introduction

The importance of hand hygiene in preventing health care associated infections (HCAIs) has been known since the landmark study carried out by Semmelweis in 1884.[1] Many studies down the ages, have clearly demonstrated effective hand hygiene to be the single most effective method in reducing HCAIs.[2–4]

Despite this, hand hygiene compliance among health care personnel has remained abysmally poor, especially in the intensive care unit (ICU).[5] This has been attributed in part, due to the poor design and quality of the information and training imparted to health care workers.[6–8] In order to tackle this problem, the World Health Organization (WHO) recently developed a concept called “My five moments for hand hygiene”.[9] It describes the fundamental reference points for hand hygiene and designates the specific moments when hand hygiene is required to effectively interrupt microbial transmission during the normal care sequence of patients.

There is very little data available on hand hygiene practices among healthcare personnel in India. Also, the level of awareness of the need for effective hand hygiene among the various sections of health care workers remains largely unknown. Thus, we decided to conduct a before–after, prospective, observational study of hand hygiene practices in our ICU, with an interventional strategy based on “my five moments of hand hygiene”.

The main purposes of our study were to assess the rates of hand hygiene compliance among health care workers, to evaluate the levels of awareness and reasons for non-compliance of hand hygiene and to study the efficacy of an interventional strategy, based on “my five moments of hand hygiene” in improving hand hygiene compliance within our ICU.

## Materials and Methods

This prospective study was conducted in the adult ICU of a tertiary care hospital in northern India. This is an open, mixed medical–surgical unit comprising of 13 beds with an average of 1300 admissions a year. The study was conducted over 6 months (November 2009 to April 2010). The initial period of observation of hand hygiene compliance was conducted over a period of 6 weeks. Here, observations on activities around individual patients were carried out in random 10-minute period intervals during the daytime, which are the busiest shifts in the ICU. A target ICU patient was selected by randomly drawing lots at the start of each observation period and was observed continuously for the entire 10-minute period. All health care personnel who contacted the target patient during this period, including doctors, visiting consultants, nurses and paramedical personnel (e.g. physiotherapists, ward helpers, radiographers, etc), were observed unobtrusively by one of the observers. During the observations, the category of the health care personnel and the compliance for each hand hygiene opportunity that presented were noted. If curtains were drawn around a bed during our observations, or for some reason the view to the patient was obscured, we discontinued observations and did not include that particular observation episode into our data. This was decided prior to the study. The tool used for observation [Table 1] and the questionnaire tool [Table 2] were both well-validated tools invented by the National Center for Patient Safety of the Department of Veterans Affairs (USA) and developed by the Veterans Affairs-3M Six Sigma Project and the Veterans Affairs “Infection: Don’t Pass it on“ campaign. These tools were downloaded from the United States Department of Veterans Affairs website (http://www.patientsafety.gov/SafetyTopics/HandHygiene/index.html). The special instruction form which accompanied the observation tool helped us to understand and standardize the tool. In our study, only two observers were involved in conducting all the observations, both during the pre-intervention as well as the post-intervention study periods. Before the start of the study period, both the researchers had discussed all aspects of observations in detail regarding what constituted each hand hygiene opportunity and what a lapse was and only the opportunities as listed on the observation sheet were recorded. We also conducted 10 trial runs of observation periods where we cross-checked each other’s observations and clarified doubts. This ensured uniformity in observations. These tools selected for our study were chosen because they were simple, clear and described each observation episode in detail. During the formal study period too, the consistency of the observations were validated intermittently by checking on selected episodes. Immediately after each observation interval by one of the other authors.

**Table 1:** Hand hygiene observation tool

Date:_____	Observed by:____________________								
Staff title										

Observations		Yes	No	Yes	No	Yes	No	Yes	No
Before clean and aseptic procedures, including medication preparation and prior to preparation, gown and glove for sterile procedures										
After contact with blood, body fluids, secretions or excretions, mucous membranes, non-intact skin										
After handling objects and devices such as soiled linen, trash, equipment									
After removing gloves used for contact with body substances										
Before patient contact										
After patient contact										
Before patient’s equipment contact										
After patient’s equipment contact										
Gloves used whenever potential for hand contact with blood/body fluids substance										
Gloves removed immediately after use to avoid contaminating the environment										

**Table 2:** Hand hygiene questionnaire

Job Function:		□ Staff nurse	□ Consultant	□ Resident	□ Physiotherapist
Date: / /					
1)	Is there a Hand Hygiene Protocol in the ICU or hospital that you are aware of?							
	□ yes	□ no	□ don’t know		
2)	If there is a protocol, what do you estimate your compliance rate at? ________							
	□ never	□ 1-10%	□ 11-40%	□ 41-70%	□ 71-100%
3)	When you don’t disinfect your hands (use soap or an alcohol hand-rub to kill microbes) when you should, what is the reason why? (Please check all that apply)							
	□ too busy	□ forget	□ unsure of need	□ out of product(s)	
	□ product(s) not in convenient location		□ other____________		
4)	To what degree do you think there is a relationship between good hand hygiene practices and preventing hospital acquired infections?							
	□ Very weak	□ Weak	□ Neither weak nor strong	□ Strong	□ Very Strong
5)	When working with another caregiver and you forget to disinfect your hands before touching a patient, what percent of the time does your colleague remind you?							
	□ never	□ 1-10%	□ 11-40%	□ 41-70%	□ 71-100%
6)	When working with a colleague who forgets to disinfect his/her hands before touching a patient, what percent of the time do you remind them?							
	□ never	□ 1-10%	□ 11-40%	□ 41-70%	□ 71-100%
7)	How often (%) do you use these products to disinfect your hands –should add up to 100%.							
	_____% soap and water alone	_____% alcohol gel or foam alone	_____% both	_____% neither	
8)	Please rate your satisfaction with the hand hygiene practices (including glove practices) currently used at your hospital.							
	□ dissatisfied	□ somewhat dissatisfied	□ neutral	□ somewhat satisfied	□ satisfied
10)	Please rate your satisfaction with hand hygiene materials currently used at your hospital.							
	□ dissatisfied	□ somewhat dissatisfied	□ neutral	□ somewhat satisfied	□ satisfied
11)	If we could do one thing to help you with practicing appropriate hand hygiene, what would it be?							

Immediately after the 6-week observation period was over, we circulated a self-report questionnaire among resident trainees, staff nurses, physiotherapists and visiting consultants who were involved with patient care in our ICU [Table 2]. The other health care worker groups (technicians, ward helpers, nursing students, radiographers) were not handed the questionnaire as their literacy level was poorer, hence their ability to read, understand and reliably answer the questions were variable. The questionnaire was aimed at evaluating the awareness and self-perception of health care workers on hand hygiene compliance and assessing the perceived barriers to the use of appropriate hand hygiene measures. The researchers themselves handed each of the questionnaires to the personnel targeted and collected them back immediately. This was to ensure that health care personnel were not influenced by other personnel. Through the questionnaire, we aimed to increase awareness on the need of hand hygiene by raising relevant questions. The responses we obtained also helped to formulate the intervention program.

This was then followed by a multimodal interventional strategy which included

1. Educational initiatives (based on “my five moments of hand hygiene”);
2. Visual reminders, in the form of posters in the common areas and beside each patient in the ICU;
3. Verbal reminders, by pointing out the lapse to health care workers whenever a breach occurred and reminding them to use the hand hygiene solutions; and
4. Ensuring easy and liberal availability of hand hygiene solutions and facilities. Hand hygiene agents were also made more conspicuous in location.

The interventional program targeted resident trainees working/visiting the ICU, visiting consultants, all nursing staff working in the ICU, physiotherapists posted in the ICU and the paramedical staff (technicians, ward helpers, radiographers, etc.) who visited our unit. The educative sessions were conducted within the ICU teaching area for nursing personnel and other technical staff working in the ICU. We held four classes for these groups to ensure that all the staff working in different shifts had equal opportunity to participate in them. Three lectures were held for the residents and consultants working in other departments (one each for surgical, medical and anesthesia departments) and these classes were conducted in the respective department lecture halls. The educational lectures were delivered by one of the three researchers conducting this study and based on “my five moments of hand hygiene”, using the same Microsoft Power Point presentation created specially for this purpose. These classes lasted approximately 1 hour and were conducted in the language that each group predominantly understood. During these classes, we drew special attention to the concept of the two zones within the ICU, the specific five moments required for hand hygiene and how to incorporate them into the natural flow of high density care in the ICU. The discussions following these classes were individualized to make them relevant to each health care worker group.

Simultaneously, eye-catching posters, including that on “my five moments of hand hygiene”, were prominently displayed in common patient areas and more detailed posters, listing reasons for using hand hygiene, were displayed in the doctors’ and nurses’ stations. At each hand-washing basin, we displayed pictures on steps of correct hand-washing procedures.

We also verbally reminded health care workers coming in contact with patients to use hand hygiene. Bedside staff nurses were especially encouraged to remind and offer hand rubs personally to visiting health care personnel who forgot to use them. Besides this, we also increased the ward supply of alcohol-based hand rubs in our unit. These were made readily available and placed prominently beside each patient.

The intervention program was completed in 8 weeks.

Following this, a 6-week repeat observation was carried out on the hand hygiene practices. The method of observation was the same as that undertaken during the pre-intervention period.

The “Hawthorne effect” is a bias that we considered might occur during the study, as the researchers involved in the intervention program were the same conducting the observations. However, there were limitations with regard to funding and time, because of which a neutral person could not be employed and trained for the observations. There were also concerns that placing such a person, who was otherwise unrelated to the work in the ICU, might raise suspicion of some kind of a study being conducted. The researchers also wanted to limit the number of people involved in the study so as to keep the observations discreet.

The categorical variables were compared by the χ^2^ test or Fisher exact test, using the epi info software (version6) and *P* < 0.05 was considered significant.

The definitions used were as follows. Hand hygiene was defined as hand washing with soap and water continuously for 2 minutes or using bedside alcohol-based hand rub solution (75% isopropyl alcohol, weight to weight ratio). The opportunity for hand hygiene was based on the concept defined under “my five moments of hand hygiene”, i.e. a) before patient contact, b) before aseptic task, c) after body fluid exposure, d) after patient contact, and e) after contact with the surroundings of the patient.

Hand hygiene was required regardless of whether gloves were used or changed.

## Results

We had 82 observation periods with 1001 opportunities for hand washing in the pre-intervention period and 90 observation periods with 1026 opportunities for hand washing in the post-intervention period in our ICU. The first observer had 48 and 52 observation periods in the pre- and post-intervention periods and the second observer had 34 and 38 observation periods in the pre- and post-intervention periods. The numbers of observation episodes were nearly similar in both the periods for each observer (i.e. 58.5 and 57.7% observations in the pre- and post-intervention periods for the first observer, and 41.5 and 42.3% observations in the pre- and post-intervention periods for the second).

**Figure 1:**
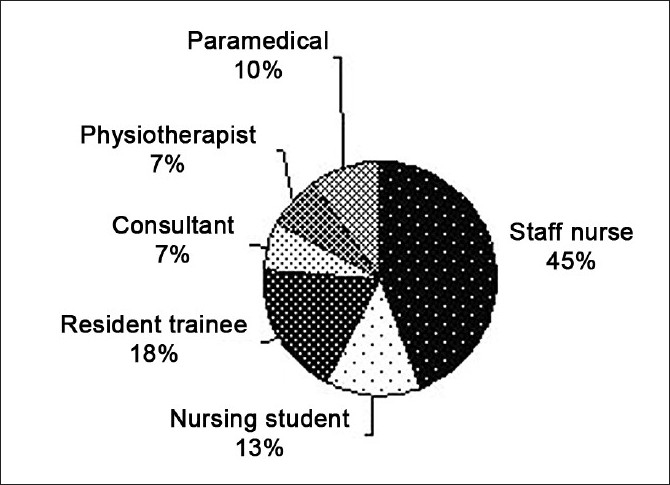
Distribution of hand hygiene opportunities (overall)

We found that bedside staff nurses involved in patient care had the maximum opportunities for hand hygiene (46.9 and 41.6% in the pre- and post-intervention periods, respectively) followed by resident trainees (18.46 and 19.1% in the pre- and post-intervention periods, respectively). Figure 1 shows the overall hand hygiene opportunities that presented to the health care workers in the pre as well as the post intervention periods. The maximum opportunities for hand hygiene were found to be in the areas of “before/after patient contact” (487 and 597 opportunities in the pre- and post-intervention periods, respectively) followed by “before/after equipment contact” (178 and 210 opportunities in the pre- and post-intervention periods, respectively).[Table 3]

**Figure 2:**
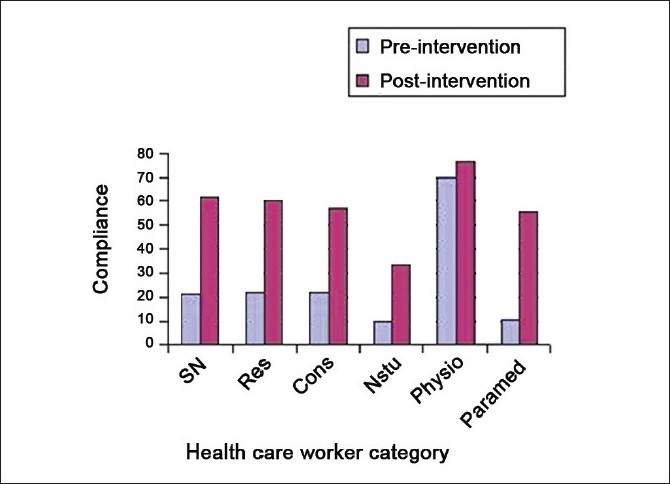
Improvement in hand hygiene compliance following intervention among health care workers in the intensive care unit

We found that the overall hand hygiene compliance in our ICU was only 25.95%, and following intervention, compliance improved significantly to 57.36% (*P*<0.0001) [Figure 2]. This difference was highly significant. Hand hygiene compliance improved among all health care worker groups [Table 4], in staff nurses from 21.48 to 61.59% (*P*<0.0000), in nursing students from 9.86 to 33.33% (*P*< 0.0000), in resident trainees from 21.62 to 60.71% (*P*< 0.0000), in visiting consultants from 22 to 57.14% (*P*=0.0001), in physiotherapists from 70 to 75.95% (*P*=0.413) and in paramedical staff from 10.71 to 55.45%, (*P*<0.0000). The overall hand hygiene compliance as noted under each observation category is given in [Table 5] and that for individual health care worker groups as observed under each observation category is given in Table 6.

**Table 3:** Hand hygiene opportunities among various health care categories in the pre- versus post-intervention period

Health care worker category	Hand hygiene opportunities (number)	
	Pre-intervention (%)	Post-intervention (%)
Staff nurses	470 (46.91)	427 (41.62)
Nursing students	142 (14.17)	123 (11.98)
Resident trainees	185 (18.46)	196 (19.10)
Consultants	50 (4.99)	91 (8.87)
Physiotherapists	70 (6.78)	79 (7.69)
Paramedical*	84 (8.38)	110 (10.72)

* Paramedical staff included technical staff, ward helpers and radiographers

**Table 4:** Comparison of hand hygiene compliance before and after intervention

Health care worker category	Compliance (%)		*P* value
	Pre-intervention (%)	Post-intervention (%)	
Staff nurses	21.48	61.59	0.0000
Nursing students	9.86	33.33	0.0000
Resident trainees	21.62	60.71	0.0000
Consultants	22	57.14	0.0001
Physiotherapists	70	75.95	0.413
Paramedical*	10.71	55.45	0.0000

* Paramedical staff included technical staff, ward helpers and radiographers

**Table 5:** Hand hygiene compliance specific to each opportunity (overall)

Indication	Overall compliance (%)		*P*value
	Pre-intervention	Post-intervention	
Before clean/aseptic procedures	7.65	38.64	0.004
After contact with blood/body fluids	32.29	55.43	0.008
After handling soiled objects	17.14	25	0.286
After removing gloves used for contact with body substances	15.47	43.06	0.001
Before patient contact	18.08	56.73	0.000
After patient contact	15.51	62.67	0.000
Before patient’s equipment contact	12.24	33.63	0.000
After patient’s equipment contact	20.59	38.95	0.000
Gloves donned whenever potential for contact with blood/body substance	70.40	85.12	0.573
Gloves removed immediately after use	27.06	47.12	0.007

**Table 6:** Hand hygiene compliance among different health care worker groups (pre- versus post-intervention)

Hand hygiene opportunities	Staff nurses pre/post in %	Residents pre/post in %	Nursing students pre/post in %	Consultants pre/ post in %	Physiotherapists pre/post in %	Paramedical staff pre/post in %
Before clean/aseptic procedures	23.68/31.81 (0.492)	22.22/100 (0.0055)	0/0 (0)	0/100 (0.333)	0/0 (0)	0/0 (0)
After contact with blood/body fluids	43.75/100 (0.123)	50/90.91 (0.098)	0/66.67 (0.119)	100/75 (0.800)	0/0 (0)	0/0 (0)
After handling soiled objects	42.86/100 (0.277)	60/0 (0)	0/33.33 (0)	0/0 (0)	0/0 (0)	0/16.67 (0.628)
After removing gloves used for contact with body subs±	16.67/58.33 (0.011)	33.33/0 (0)	0/0 (0)	0/71.42 (0)	42.86/100 (0.27)	0/28.57 (0.381)
Before pt* contact	10/68 (0.000)	9.43/61.54 (0.000)	0/25 (0.001)	19.05/37.5 (0.152)	70/77.78 (0.49)	0/70.59 (0.000)
After pt contact	16/65.22 (0.000)	14.81/60.32 (0.000)	5.13/43.75 (0.000)	23.81/62.5 (0.005)	28.57/84.21 (0.013)	4.76/60 (0.000)
Before pt’s equipment contact	2.04/48.98 (0.000)	0/44.44 (0.005)	0/25 (0.065)	0/0 (0)	71.43/33.33 (0.208)	0/50 (0.238)
After pt’s equipment contact	2.13/51.06 (0.000)	21.43/57.89 (0.036)	0/27.78 (0.060)	50/0 (0.500)	50/33.33 (0.547)	0/63.64 (0.006)
			
Gloves worn when potential for contact with blood/body subs±	78.72/82.14 (0.720)	62.5/28.57 (0.148)	92.3/100 (0.812)	0/100 (0.017)	100/100 (0)	88.89/100 (0.818)
Gloves removed imm† after use	33.33/54.17 (0.1088)	44.44/57.14 (0.500)	0/0 (0)	0/100 (0)	84.61/71.43 (0.361)	0/0 (0)

* Pt – patient,

± subs – substance

† imm – immediately, All values in percentages

*P* value indicated in parenthesis

**Figure 3:**
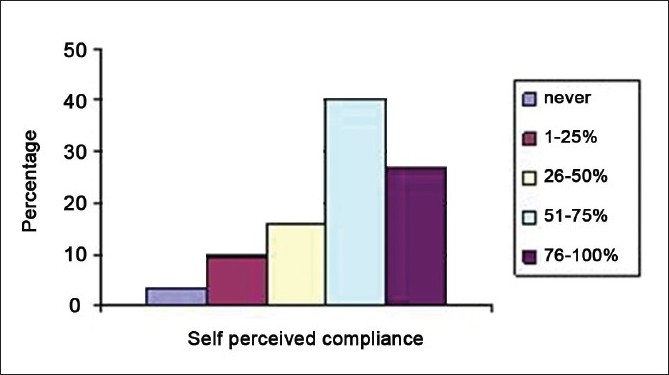
Self-perceived compliance of hand hygiene among health care workers in the intensive care unit

**Figure 4:**
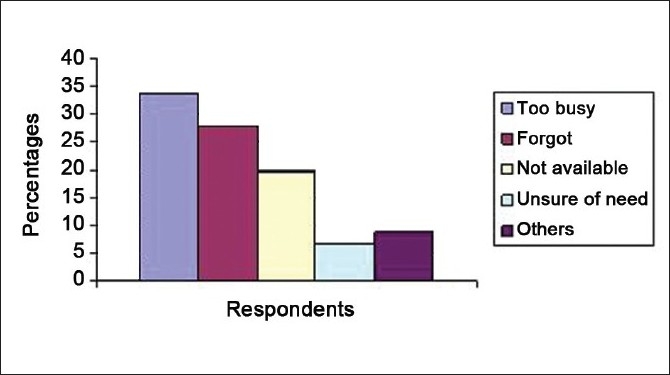
Reasons for not using hand hygiene among health care workers in the intensive care unit

Prior to the intervention strategy, a 12-point self-report questionnaire was distributed among 105 health care workers who were involved with patient care in our ICU [Table 2]. This included 24 staff nurses, 36 resident trainees, 8 consultants, 31 physiotherapists, and 6 ICU technicians. All the questionnaires distributed were collected immediately and available for analysis. On analysis, it was seen that 91.4% of the respondents were aware of an ICU protocol on hand hygiene in our unit. The self-perceived rates of compliance with hand hygiene were quite high. Sixty-seven percent estimated their hand hygiene compliance rate at more than 50% of the time [Figure 3] and the most frequent reason for lack of compliance was that they were too busy (33.7%) [Figure 4].

Though 95% of the respondents agreed that there was a strong/very strong association between hand hygiene and preventing HCAIs, few (approximately 25%) of them were reminded of using the hand hygiene product by a colleague. Only 13% of the respondents, in turn, reminded their colleagues of the need to use a hand hygiene product at 40% or more opportunities. More than half of the respondents were satisfied/somewhat satisfied with the hand hygiene practices in the ICU (55.6%) and with the hand hygiene products currently being used in the ICU (64.7%).

When we compared the responses received with the actual data observed, we found that the self-perceived rates of hand hygiene compliance were much more than the actual rates. While about 67% of health care workers estimated their hand hygiene compliance as more than 50%, the actual observed compliance in the pre-intervention period was only 23% [Table 7]. Table 8 lists the suggestions by respondents to help increase the hand hygiene compliance in our unit.

During the intervention period, the educational sessions reached 72.5% of resident trainees, 82% of bedside staff nurses, 95.3% of physiotherapists, 30.33% of visiting consultants and 45.7% from among the paramedical staff group. The visiting consultants were the most difficult to reach with this intervention, probably because they felt that they “knew” all about hand hygiene and did not need to attend these sessions.

The other interventions carried out were visual reminders (Posters) displayed prominently in the ICU, verbal reminders to all health care personnel involved and ensuring ample and easy availability of hand hygiene products in the unit. It was difficult to quantify the population reached with these modalities as their effect continued day and night, influencing staff and visitors to the ICU.

**Table 7:** Self-estimated hand hygiene compliance among health care workers versus actual compliance rates: Most health care workers overestimated their compliance rates

	Self-estimated hand hygiene rates (% compliance rates)			Observed health care workers compliance rates (%)
	25–49%	50–74%	75–100%	
Resident trainees	13.9	41.66	16.67	21.62
Staff nurses	25	50	24	21.48
Consultants	12.5	25	25	22
Physiotherapists	16.1	32.2	45.1	70

**Table 8:** Suggestions from health care workers to improve hand hygiene compliance in the intensive care unit

Suggestions	Percentage of respondents
Improve disinfectant quality/availability	27.88
Provide educational sessions	14.5
Verbal reminders	10.57
Visual reminders	6.7
Ensure stricter enforcement of hand hygiene in the unit	4.8
Provide incentives to those complying	2
No suggestions	33.5

## Discussion

A large proportion of the infections acquired in the ICU have been attributed to cross contamination and transmission of microbes from hands of health care workers to patients.[10,11] Many studies have consistently shown that improved hand hygiene practices have reduced nosocomial infections and cross transmission of multidrug resistant infections in hospitals.[9,12–15] Despite this, present day data suggest that hand hygiene compliance among health care personnel in most hospitals is at best, less than 50%.[13] Working in busy wards (ICUs), doctors (as compared to nursing personnel), understaffing, overcrowding, high-intensity patient care, insufficient time, lack of institutional priority, etc. were some of the risk factors found to be associated with poor hand hygiene compliance. Many attempts have been made in the past to improve hand hygiene compliance, such as educational interventions, motivational programs, etc. However, most of these met with little or temporary success. Hence, several multifaceted interventions, which include behavioral, environmental and social changes, have been suggested and tried to sustain improvements in hand hygiene compliance.[10,16]

In the present study, we purposed to observe the hand hygiene practices among health care workers in our ICU to evaluate the reasons for noncompliance and to study the impact of multiple interventions including an educative strategy based on “my five moments for hand hygiene”, in improving hand hygiene compliance in our unit.

During the first phase of our study, we found that the overall hand hygiene compliance in our ICU was approximately 26% only. Bedside staff nurses and resident trainees working in the unit had the maximum opportunities for hand hygiene in the ICU and compliance rates were low (approximately 21.5%) in both groups. We also found that hand hygiene compliance was the least among nursing students and the paramedical staff (10%). Compliance rates were very good among the physiotherapists posted in the ICU (70%). The reported hand hygiene compliance rates in ICUs vary from 35 to 80% with high rates of variability reported between the categories of health care workers, the intensity of work in the unit and the type of ICUs studied.[5,10,14,17,18]

During both the observation periods, the opportunities for hand hygiene were most in the areas of “before/after patient contact” or “before/after equipment contact”, while the compliance rates specific to these opportunities were among the least (13 and 16%, respectively). Several studies have reported that the activities that generate maximum opportunities for hand hygiene were associated with least compliance rates.[13,14,16,19] Thus, incorporating hand hygiene as a part of the patient care sequence, especially in high-density work areas like the ICU, cannot be overemphasized.

In our initial observations, we noted that many health care workers wore gloves when there was likelihood for contact with blood or body fluids, but unfortunately forgot to remove them soon after and continued performing other ICU care activities with the same pair. Though wearing gloves when indicated represents compliance with hand hygiene, failure to remove them immediately after use constitutes noncompliance. In a study by Doebbeling *et al*, organisms were cultured from 4 to 100% of the gloves used and observed counts were up to 4.7 log on hands after glove removal.[20] Our intervention strategy emphasized the need to remove gloves as soon as the indication was over and use hand hygiene measures after removal. We encouraged health care personnel to use visual cues (like linking the act of glove removal with the need to use hand rub) to remind themselves of this. In the post-intervention period, glove disposal rates improved significantly (from 27 to 47%).

Certain additional observations were made during our study. We found that, at times, though nursing staff and resident trainees used hand hygiene as indicated, they unconsciously touched areas of their own body or their clothes before patient contact, thereby negating the effect of hand hygiene. Lam *et al*, also noted that health care workers tend to recontaminate their hands by touching inanimate objects, pens or fomites after hand hygiene.[21] Hence, attention was drawn to this fact during the intervention sessions and personnel were shown how to consciously avoid touching other objects between hand hygiene and patient care.

The questionnaire distributed among health care workers revealed that there was a large disconnect between the perceived rates of hand hygiene compliance and actual observed rates. Most health care worker groups estimated their hand hygiene compliance as nearly 50% of the time which contrasted sharply with the actual observed rates (24.95%). The most common reason cited by health workers for noncompliance was that they were too busy (33.7%). Hence, we emphasized to our health workers how hand hygiene takes up little time and the benefits produced far outweighed the time lost in applying hand hygiene. Most respondents were aware of the importance of hand hygiene but few took the initiative to remind their co-workers of the same. A similar survey, in the form of a self-report questionnaire, was conducted by Pittet *et al*, among physicians of a large university hospital to assess their beliefs and perceptions regarding hand hygiene. They found that most physicians were aware of a risk of cross transmission of infection from lack of hand hygiene and intended to adhere to hand hygiene. Though many (65%) had a good knowledge of indications, 67% perceived hand hygiene as a difficult task.[19]

In our study, a multimodal interventional strategy was employed, with intensive educational sessions based on “my five moments of hand hygiene” as well as displaying posters, providing verbal reminders and ensuring easy and ample supply of hand hygiene products in the unit. We particularly encouraged bedside staff nurses to repeatedly remind health care personnel visiting the ICU to use hand hygiene products during the five designated moments. With these strategies, most health care workers who visited the ICU were reached. A review of literature suggests that single intervention programs produce less success in leaving a lasting impact on hand hygiene compliance.[22,23] Multimodal interventional strategies, which include audits, performance feedbacks, education, memos, posters and films, ensuring easy availability and supply of alcohol-based handrubs and strategies aiming to improve accessibility to hand hygiene agents, have been more successful.[10,14,21,22,24,25] To be successful, interventions must address individual factors, interactions within a group and within an institution.[26,27]

The intervention strategy employed by us had a significant impact in improving hand hygiene compliance rates in almost all the categorical variables studied, especially in the areas of “before” and “after patient contact”. The physiotherapists working in the ICU had good hand hygiene compliance rates even in the pre-intervention period (70%), which was probably the reason why the intervention did not seem to have a significant impact on them (75.94%, *P*= 0.413).

In a study by Lam *et al*, the hand hygiene compliance before and after the implementation of a multimodal implementation program in a neonatal ICU improved from 40 to 53% before patient contact and from 39 to 59% after patient contact. There was a more marked improvement for high-risk procedures and HCAIs fell from 11.3 to 6.2 days during the study period. They concluded that an effective education program could improve hand hygiene compliance.[21] In another study conducted in five adult ICUs, an intervention strategy consisting of educational program and improving standards of catheter care resulted in a significant decrease in catheter-related blood stream infection rates, with an increase in hand hygiene compliance from 59 to 65%.[28]

We found the concepts given in “my five moments of hand hygiene” easy to understand and teach. The notable strengths of this concept were the following.

1. Its simplicity, i.e. only five key points to remember. The concept integrated all the indications for using hand hygiene during the sequence of health care delivery into a compact “five-moment” concept.
2. Its strong visual message (display of five arrows at the five moments of hand hygiene). The single patient in the center with the two zones and the five moments for hand hygiene action arranged around the patient were visually appealing and conveyed the entire concept in a single picture.
3. Easy recollection, especially for semi-literate health care workers involved in patient care in our ICU, such as ward helpers, technicians, etc. We found that health care workers could easily remember and reproduce the five designated moments.

We believe that our intervention was successful because the program was multimodal, based on an easy to understand model and was fully supported by every member of the unit.

There were certain limitations in our study. There might have been variations in hand hygiene practices during other times like evening or night shifts, which we did not study. In a study on the diurnal variation in hand hygiene compliance in an ICU in northern India, the overall rates of hand hygiene compliance was found to be 59.9%. It was also found that hand hygiene compliance dropped during the night from 66.1 to 46% for doctors, from 60.7 to 55% for nurses and from 38.6 to 31% for paramedical staff.[29]

Another area of limitation was in matching of the subjects. While the health care personnel studied in the pre- and post-intervention period belonged to the common group of personnel targeted during the intervention period, they were not matched. The personnel studied during the two periods belonged to the same cohort, and hence, the interventions targeted all personnel belonging to each of the concerned departments.

It is possible that some health care worker groups may not have been reached through the educational interventions and the investigators hoped to disseminate information to such personnel through the other health workers who had been covered at the intervention period. There were also other issues like language barriers and varying educational levels of different staff groups which might have influenced the understanding of the need for effective hand hygiene. The other area of error might have been regarding differences in the understanding of the questions in the questionnaire circulated among various health care workers, which might have introduced some errors. The “Hawthorne effect”, as discussed earlier (in the section “Subjects and Methods”) might have introduced some bias into the study. Another limitation is that the post-test study was conducted immediately after the intervention period; however, we are planning a repeat follow-up study at 6 and 12 months to assess the long-term effects of our interventional strategy.

Our study assumes special importance as, to the best of our knowledge, there are no published data from India, on interventional strategies conducted to systematically observe and achieve an improvement in hand hygiene compliance among various health care personnel in the ICU. In our study, different categories of personnel were individually studied, targeted and their compliance assessed. Perceptions regarding hand hygiene and awareness levels were also revealed during the study.

We plan to continue the interventions used at regular intervals, to keep up the improved compliance achieved. Avenues of future research would include conducting further studies on hand hygiene to demonstrate reduction in HCAIs, as well as reduced morbidity and mortality in our ICU.

We conclude that the hand hygiene practices are abysmally low among most health care personnel working in the ICU. However, intervention strategies such as the one we employed had a good impact in improving compliance in our ICU and these improvements were consistently seen among almost all health care worker groups in our unit. The results we achieved can be easily duplicated in other ICUs across the country if similar, interventional strategies are employed.
